# Influence of cheese making process on STEC bacteriophage release

**DOI:** 10.3389/fmicb.2023.1270346

**Published:** 2023-09-28

**Authors:** Nicola Mangieri, Rui P. Vieira, Claudia Picozzi

**Affiliations:** ^1^DeFENS, Department of Food, Environmental and Nutritional Sciences, Università Degli Studi di Milano, Milano, Italy; ^2^Instituto de Medicina Molecular, Faculdade de Medicina, Universidade de Lisboa, Lisboa, Portugal

**Keywords:** prophage induction, Shiga toxin-producing *E. coli*, stx-phages, qPCR, cheese production

## Abstract

Shiga toxin-producing *Escherichia coli* (STEC) are foodborne pathogens implicated in diseases including hemolytic uremic syndrome (HUS) and hemorrhagic colitis (HC). The main virulence factor are Shiga toxins; their production and secretion are by-products of the expression of late genes of prophages upon sub-lethal environmental stimuli exposure. Hence, the lysogenic prophage after a stress switch to lytic cycle spreading the Stx phages. In the present study, 35 STEC were screened for the presence and the ability to release Shiga toxin-encoding bacteriophages. Three bacterial strains showed signals of prophage presence both in plate and in PCR. Subsequently, these bacterial strains were subjected to stressors that simulate cheese manufacturing conditions: NaCl (1, 1.5 and 2% w/v), lactic acid (0.5, 1.5 and 3% v/v), anaerobic growth, pasteurization (72°C for 15 s), UV irradiation. The ability to release prophage was evaluated by Real Time qPCR. Induction of the prophages showed that the addition of NaCl at 1.5 and 2% significantly increased viral release compared to control. Conversely, the addition of lactic acid had a significant repressive effect. The other applied stressors had no significant effect in phage release according to the experimental conditions adopted.

## 1. Introduction

*Escherichia coli* is a commensal bacterium of the gastrointestinal tract of humans and warm-blooded animals, but is also present in water and soil. In most cases these bacteria are harmless to their host, but some strains are characterized by the presence of virulence traits that can cause disease in humans (Croxen et al., 2013). Among the pathogenic strains, Shiga toxin-producing *E. coli* (STEC) is the main group involved in foodborne illness (Castro et al., 2017) causing gastrointestinal symptoms and bloody diarrhea. The disease can potentially progress into hemorrhagic colitis (HC) and hemolytic uremic syndrome (HUS) (Yang et al., 2017).

In 2020 STEC resulted the fourth most frequent bacterial agent detected in foodborne outbreaks in the EU, with 34 outbreaks, 208 cases, 30 hospitalizations and 1 death (The European Union One Health 2020 Zoonoses Report, 2021). The main reservoir of STEC are ruminants, particularly cattle, but also sheep and goats and less frequently other animals such as pigs, horses, domestic poultry, dogs, cats, and other wild animals (Kim et al., 2020). Direct contact with contaminated water or animals is also a relevant route of transmission to humans (Koutsoumanis et al., 2020).

As concern prevalence in food, STEC was most commonly found in meat of different types derived from different animal species (3.4% STEC-positive) and in milk and dairy products (2.1%), while fruits and vegetables were the least contaminated products (0.1%) (The European Union One Health 2020 Zoonoses Report, 2021).

Several virulence factors, including intimin encoded by *eae* gene, are involved in enteropathogenic and enterohaemorrhagic diarrhea. Shiga toxins, for which encoding genes are carried by bacteriophages located in the bacterial chromosome, are the main cause of virulence. Bacteriophages can be divided in lytic and temperate according to their growth potential. Two types of life cycle can occur: lytic and lysogenic. In the lytic cycle, the bacteriophage injects its nucleic acid into the bacterial host and replicates, taking control of the host’s molecular machinery. Then, phages lyse the bacterial cell with the production of two types of protein: holins and lysins. The first works to perforate the bacterial cytoplasmic membrane, giving the lysins access to bacterial cell wall (Cisek et al., 2017; Kakasis and Panitsa, 2019). Shiga toxins are expressed during the lytic cycle of bacteriophages that carry the genes for these toxins. During the lysogenic cycle, after the injection phase, the phage nucleic acid integrates into the host bacterial chromosome and replicates along with generations by vertical gene transfer. The expression of the phage genes, including those for Shiga toxins, is repressed during lysogeny. However, when the lysogenic cycle is induced, such as by exposure to stress factors as changes in pH, presence of iron, presence or absence of ions, presence of antibiotics or DNA damage, the phage DNA excises from the host chromosome and enters the lytic cycle (Lin et al., 2017). This results in the production of new phage particles, and the expression of phage genes, including those for Shiga toxins (Rodríguez-Rubio et al., 2021). Therefore, while Shiga toxins are not expressed during the lysogenic cycle, they can be expressed during the lytic cycle of Stx phages. As written before, antibiotics have been reported as prophage inducers. Indeed, in the clinical treatment of STEC infections the use of antibiotics is controversial due to the release of prophages that can infect non-STEC strains and lead to overproduction of toxins (McGannon et al., 2010).

The lysogenic phages involved in the transmission of the Shiga toxin are called Stx phages. Shiga toxin is an exotoxin produced only by STEC and *S. dysenteriae* serotype 1 and is characterized by an AB5 structure containing an A subunit that is not-covalently associated with the five B subunits (Yang et al., 2017). The A subunit plays a role in inhibition of protein synthesis and in cell damage by apoptosis (Yang et al., 2015) and the five B subunits bind the globotriaosylceramide receptor (Gb3) on the surface of susceptible eukaryotic cells. After assimilation by endocytosis, the toxin pass through the trans-Golgi network and endoplasmic reticulum and then hinders ribosomal function by cleavage of adenosine residues from the 28S rRNA of the large subunit (Pacheco and Sperandio, 2012).

STEC strains are a great concern to the dairy industry since they can cause diseases in humans even with a small number of ingested cells (<10) (Etcheverría and Padola, 2013). Furthermore, several psychophysical factors in cheese making process, such as addition of NaCl and changes in temperature and in pH can be considered as potential stressors to induce bacteriophage release and to promote toxin transmission among bacterial cells. Milk pasteurization prevents the possible STEC contamination, but some cheeses are made using raw milk. Besides, STEC can contaminate the product during the different manufacturing and processing steps and persist in the final product (dos Santos Rosario et al., 2021).

The objectives of this work were to characterize temperate bacteriophages from STEC strains used in this work and to evaluate the influence of stress factors related to the cheese-making process, on induction phenomena through a quantitative Real-time PCR (qPCR).

## 2. Original research

### 2.1. Materials and methods

#### 2.1.1. Bacterial growth conditions

The STEC strains used (Table 1) are part of Department of Food, Environmental and Nutritional Sciences Collection from Università degli Studi di Milano and were partially characterized in previous works (Picozzi et al., 2016; Mangieri et al., 2020). Strains were streaked on Tryptone Bile X-Glucuronide agar (TBX) (Merck, Darmstadt, Germany) plates and incubated at 37°C for 24 h. A single colony was transferred in a 10 mL LB broth (Alfa Aesar, Karlsruhe, Germany) tube and incubated overnight at 37°C. The overnight culture was centrifuged at 1200 *g* for 15 min (Centrifuge 5,415 D, Eppendorf, Hamburg, Germany), the supernatant was discarded, and the pellet was resuspended in LB broth with 20% (v/v) glycerol and stored at −20°C until further use.

**Table 1:** List of STEC strains.

Strain	Sample source	Serogroup
214CH	Human stool	O157
214R-ACH	Human stool	O26
214R-MCH-B	Human stool	O157
224SMA-GS	Human stool	ND
225R-A	Human stool	O26
226BB	Human stool	O157
227MCH	Human stool	O157
227Rosa	Human stool	ND
228GS	Human stool	O145
229B-ACH	Human stool	ND
229 M-AS	Human stool	ND
229PRAL-ACH	Human stool	O26
229PRAL-AS	Human stool	ND
229RACH	Human stool	O111
229Rosa-A	Human stool	ND
231PCH-A	Human stool	ND
232AS-B-LUC	Human stool	ND
233P-CH-A	Human stool	ND
239R-A	Human stool	ND
242CH	Human stool	O157
242Rossa	Human stool	O157
243RACH	Human stool	O26
L12-2	Raw Goat Milk	O26
L36-2	Raw Goat Milk	ND
F1-1	Goat’s Milking Filter	O26
F10-4	Goat’s Milking Filter	O26
F80-1	Goat’s Milking Filter	ND
F80-2	Goat’s Milking Filter	ND
F80-3	Goat’s Milking Filter	ND
F80-4	Goat’s Milking Filter	ND
F90-1	Goat’s Milking Filter	ND
F90-3	Goat’s Milking Filter	ND
F93-3	Goat’s Milking Filter	O26
F95-2	Goat’s Milking Filter	O26
F95-3	Goat’s Milking Filter	O26

### 2.2. Bacteriophage DNA extraction and PCR analysis

Strains were subjected to phage induction by adding 0.3 mg/mL of Fosfomycin (Sigma-Aldrich, St. Louis, United States) to cells at exponential phase (OD600nm = 0.2–0.3). After 6 h of incubation at 37°C, the solutions were centrifuged at 4800 *g* for 10 min (Centrifuge 5,415 D, Eppendorf, Hamburg, Germany) and filtered through a 0.45 μm membrane filters (Minisart Syringe filter). The crude filtrates were tested for the presence of phages *via* spot-test in LB agar plates using two sensitive *E. coli* strains CNCTC 6896 and CNCTC 6246 as previously described (Mangieri et al., 2020). Briefly, the exponentially growing CNCTC 6896 and CNCTC6246 cultures (100 μL) were supplemented with CaCl_2_ to a final concentration of 10 mM and mixed with melted LB soft agar (4 mL) at 48–50°C in sterile test tubes. Each mixture was then poured onto LB bottom agar (1.5%) plates to create a double layer. Phage filtrates (10 μL) were spotted onto agar surface and plates were incubated overnight at 37°C. The presence of infected phage particles was confirmed by the appearance of clear bacterial lysis.

Subsequently, 50 mL of crude bacteriophage filtrate were precipitated by addition of 10% (w/v) of polyethylene glycol (PEG) 6,000 (Merck, Darmstadt, Germany) and 0.5 M NaCl. After 6 h at 4°C, the solution was centrifuged at 4800 *g* for 10 min (Centrifuge 5,415 D) and the supernatant discarded. The pellet was resuspended in 400 μL of SM buffer (100 mM NaCl, 8 mM MgSO_4_, 50 mM Tris–HCl, pH 7.5, 0.01% gelatin) and incubated overnight at 4°C.

The suspension was subjected to an enzymatic treatment with 5 μL of DNase (20 mg/mL) (Roche, Mannheim, Germany) and 10 μL of RNase (5 mg/mL) (Merck, Darmstadt, Germany) at 37°C for 60 min to remove any non-phage nucleic acids. Subsequently, the enzymes were inactivated by heat treatment at 75°C for 10 min.

A PCR amplification of the 16S rRNA gene was performed to verify the absence of bacterial DNA using the universal primers BSF-8 (5’ AGAGTTTGATCCTGGCTCAG 3′) and BSR-1541 (5’AAGGAGGTGATCCAGCCGCA 3′). The PCR products were processed by electrophoresis and, in case no bacterial DNA was detected, the extraction process continued.

Then, 50 μL of EDTA (0.5 M; pH 8), 50 μL of SDS 10% (w/v) and 2.5 μL of Proteinase K (20 mg/mL) were added to the phage suspensions followed by incubation at 37°C for 1 h. Subsequently, 400 μL of saturated phenol were added, gently mixed, and centrifuged at 13400 *g* for 10 min (Centrifuge 5415 D). The aqueous phase was transferred to a new tube and 200 μL of saturated phenol and 200 μL of chloroform: isoamyl alcohol (24:1) were added and centrifuged at 13400 *g* for 10 min. The liquid phase was transferred in a new tube and added with 200 μL of sodium acetate (3 M; pH 7) and 600 μL of isopropanol, for DNA precipitation. After centrifugation under the same condition, the pellet was resuspended in 200 μL of Ethanol 70% (v/v), followed by centrifugation at 13400 *g* for 4 min. Ethanol was then discarded and pellet dried. After drying at 37°C for 60 min, the pellet was resuspended in 50 μL of TE buffer (Tris–HCl 10 mM, EDTA 1 mM, pH 8) and the DNA was stored at −20°C until further usage.

Phage DNA was tested for the presence of *stx* (*stx1, stx2a, stx2f*) and *eae* genes by PCR, according to EU-RL VTEC_Method_01_Rev 0 (2013) protocol (European Union Reference Laboratory (EURL), 2013). DNA from *Escherichia coli* O157:H7, carrying *stx1*, *stx2* and *eae* genes, was used as positive control in PCR. The previously isolated lytic bacteriophage FM10, not carrying virulence genes, was used as negative control (Mangieri et al., 2020).

### 2.3. Assessment of bacteriophage inducers by qPCR

STEC strains at the beginning of the exponential phase (OD600nm = 0.2–0.3) were subjected to 0.5, 1.5 and 3% (v/v) lactic acid, 1, 1.5 and 2% (w/v) NaCl, anaerobic growth in LB tubes, pasteurization at 72°C for 15 s, UV irradiation (20 cm distance for 60 s), as stress factors related with cheese production.

After incubation at 37°C for 16 h, the samples were centrifuged at 4800 *g* for 10 min (Centrifuge 5,415 D) and filtered through 0.45 μm filters (Minisart® Sartorius). To remove bacterial DNA, 100 μL aliquots were treated with DNase and RNase (10 mg/mL each) at 37°C for 30 min followed by heat treatment at 70°C for 10 min to inactivate the enzymes.

The assay was designed for 15-μl reactions (QPCR Green Master Mix LRox 1X, Biotechrabbit, Hennigsdorf, Germany) containing 400 nM of primers stx1F (5’ATAAATCGCCATTCGTTGACTAC 3′) and stx1R (5’ AGAACGCCCACTGAGATCATC 3′). Real-time qPCR assays were carried out in a MasterCycler® ep Realplex (Eppendorf AG) with an initial denaturation at 95°C for 3 min and 40 cycles as follows: 95°C for 15 s, 60°C for 30 s, 65°C for 30 s. A standard curve was obtained by 5-point interpolation of 10-fold serial dilutions of a bacterial gDNA extracted from STEC strain 225R-A carrying the *stx1* gene (Supplementary Figure S1). The DNA concentration was measured through a spectrophotometric lecture at 260 nm, and the DNA copy number was calculated using a ThermoFisher tool: “DNA Copy Number and Dilution Calculator.”

Each experiment was replicated 4 times for each strain. The analysis of variance with post-hoc Tukey HSD (Honestly Significant Difference) was performed using the open-source software: R Core Team (R Core Team, 2017), with the package “agricolae,” “ggplot2” package for graphic processing.

### 2.4. Evaluation of bacterial growth after different stressors

To assess cell growth of STEC subjected to different stressors, 200 μL of each bacterial strain were transferred in a 96-wells plate and incubated at 37°C for 16 h, monitoring the optical density (OD) at 600 nm every 15 min through a plate reader (PowerWave XS2, BioTek, Winooski, VT, USA) using Gen5 software. Only samples submitted to oxygen deprivation were transferred into the plate and analyzed at the end of the incubation. Each experiment was performed in triplicate.

## 3. Results

### 3.1. Bacteriophages virulence gene assessment

**Figure 1:**
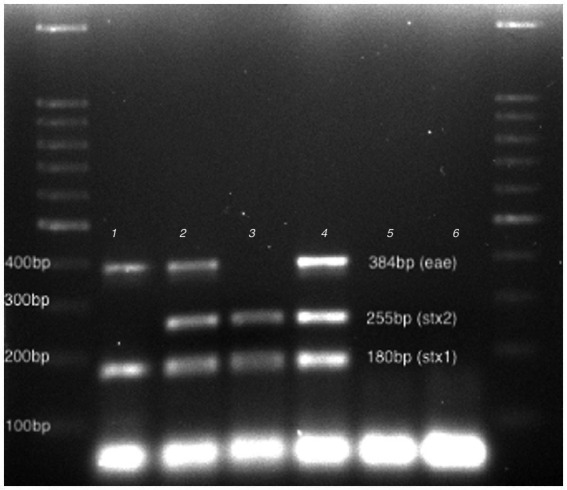
Virulence profiling. 100 bp DNA Ladder (LeGene Biosciences, San Diego, United States), 1: vB_Eco225R-A, 2:vB_Eco229R-ACH, 3: vB_EcoF1-1, 4:ATCC 35150 (positive control), 5. FM10 (negative control.), 6: no template and DNA Ladder.

To select the bacterial strains able to release *stx*-phage after a stress, 35 STEC strains were subjected to phage induction using Fosfomycin. Three strains (225R-A, 229RACH and F1-1) showed a sign of bacterial cell lysis in LB double layer agar plates (Supplementary Figure S2) and the presence of *stx* and *eae* genes after PCR amplification of filtrates. In particular, the *stx1* gene was found in all three phages filtrates, the *stx2* was detected only in the 229RACH and F1-1, the *eae* gene in 225R-A and 229RACH (Figure 1). The phage particles were then named vB_Eco225R-A, encoding *stx1* and *eae*, vB_Eco229R-ACH, encoding *stx1*, *stx2* and *eae* and vB_EcoF1-1, encoding *stx1* and *stx2*, based on the nomenclature scheme proposed in Kropinski, Prangishvili, & Lavigne (Kropinski et al., 2009).

### 3.2. Influence of stressors on bacteriophage induction

The STEC strains that host the bacteriophages have been exposed to various stressors related to the cheese-making process: sodium chloride at three different concentrations (1, 1.5 and 2% w/v), lactic acid (0.5, 1.5 and 3% v/v), pasteurization, UV irradiation and oxygen deprivation.

Evaluation was performed by Real Time qPCR amplification of the *stx1* gene, based on the common presence of this gene in the three lysogenic bacteriophages used in this work. To quantify the amount of DNA contained in the samples, a 5-point standard curve was used: the linear relationship of Ct versus Log (copies/mL) was Y = −3,496x + 43,068, R2 = 0,9,983.

The results, summarized in Table 2, showed that phage induction increased in direct proportion to the NaCl concentration. In fact, the average values were: 8.19, 7.97 and 7.15 Log DNA copies/mL for the addition of 2, 1.5 and 1% of NaCl, respectively, against a spontaneous induction of 6.49 Log DNA copies/mL. Increasing the salinity level to 1.5 and 2% (w/v) significantly boosted the viral content. The effects of heat treatment (72°C for 15 s), irradiation by exposure to UV light and oxygen deprivation were investigated by quantifying phage release after 16 h incubation at 37°C. UV irradiation increased phage induction with a mean value of 7.43 compared to spontaneous induction of 6.49 Log DNA copies/mL. Furthermore, oxygen scarcity improved phage release with a mean value of 6.69 Log DNA copies/mL. In both cases, no significant differences were found (*p* < 0.05) (Table 2). Heat treatment slightly reduced spontaneous release with no significant differences (Table 2). After this treatment, the inoculated bacteria in exponential phase were eliminated. The phenomenon was confirmed by the fact that no colony grew after plating in TBX agar plates after 48 h incubation at 37°C.

**Table 2:** Summary of SYBR qPCR results organized by stressors.

Stressors	Average Log_10_ copies/mL	std	*n*	groups
2%NaCl	8.19	0.91	12	a
1.5%NaCl	7.97	0.74	12	ab
UV	7.43	0.88	12	abc
1%NaCl	7.15	0.94	12	bcd
Ox	6.69	0.72	12	cd
Sp	6.49	1.00	12	cd
T°	6.29	0.95	12	d
LacAc	5.30	0.32	36	e

Values with different letters are significantly different groups (*p* < 0.05) assigned by One-way ANOVA (Analysis Of Variance) with post-hoc Tukey HSD (Honestly Significant Difference). Ox: oxygen deprivation; Sp: spontaneous release; *T*, heat treatment; LacAc, Lactic acid; std, standard deviation, *n*, number of samples.

To mimic the stressful conditions that protein coagulation can inflict on STEC during the transformation of milk into cheese, caused by the pH-lowering activity of lactic acid bacteria metabolism, the strains were exposed to lactic acid (0.5, 1.5 and 3% v/v). Growth is highly dependent on pH. The data reported in Supplementary Table S1 showed a substantially homogeneous result in the three lactic acid additions (0.5, 1.5 and 3%v/v) performed. Thus, lactic acid addition per strain was reported as lactic acid addition considering the 12 repetitions for each bacterial strain used, regardless the amount of acid added (Table 2).

Noticeably, prophage release was significantly reduced by lactic acid stress, with an average of 5.30 compared to spontaneous release of 6.49 (*p* < 0.05) Log DNA copies/mL.

Considering the single phage behavior under all the stress conditions applied, the average values were 7.35, 6.36 and 6.12 Log DNA copies/mL for the strains 225R-A, F1-1 and 229RACH, respectively. The 225R-A strain was significantly different from the other two (p < 0.05) (Table 3).

**Table 3:** Summary of SYBR qPCR organized by different strains.

Bacteria	Average Log copies/mL	std	*n*	groups
225R-A	7.35	1.41	40	a
F1-1	6.36	1.00	40	b
229RACH	6.12	1.02	40	b

Values with different letters are significantly different groups (*p* < 0.05) assigned by One-way ANOVA (Analysis Of Variance) with post-hoc Tukey HSD (Honestly Significant Difference). Copies: Log phage DNA copies/ml; Std, standard deviation; *n*, number of replicates; groups: assigned by Tukey HSD test.

**Figure 2:**
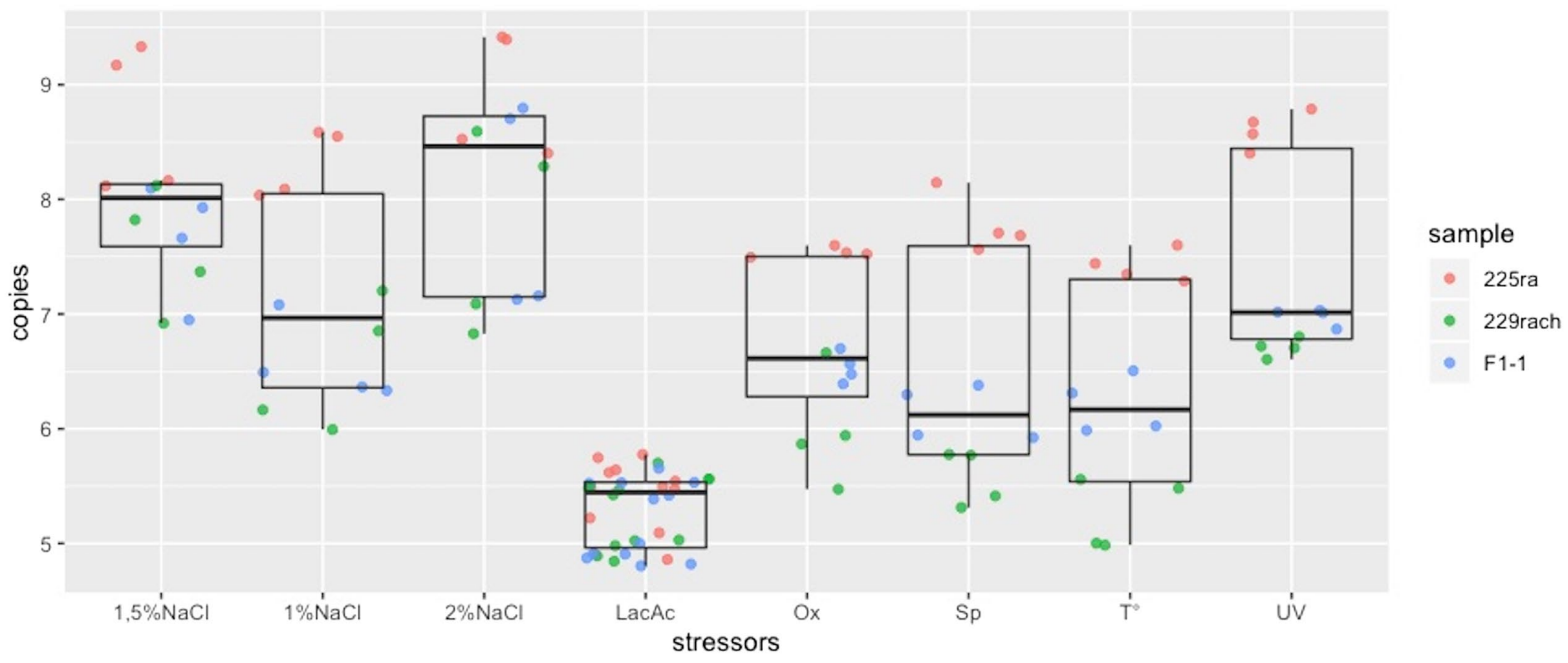
Box plots representing the distribution of the phage DNA copies/ml for each stressor. LacAc, Lactic acid; Ox, oxygen deprivation; Sp, spontaneous release; *T,* heat treatment.

Results are summarized in Figure 2 where it can be noticed that the median of spontaneous release is lower than the median of 2, 1.5, 1% NaCl, UV irradiation, heat treatment and oxygen deprivation. Hence, these stressors improve phage release.

Significant differences from the control were found for the addition of 2 and 1.5% NaCl with the improvement of phage release and a significant decrease due to the addition of lactic acid (Table 2) (*p* < 0.05). No significant differences were found for the other applied stressors.

### 3.3. Assessment of bacterial growth

To investigate how different stressors affect bacterial growth, cells were monitored through OD_600nm_ measurements every 15 min. The results are reported in Supplementary Figure S3. The data obtained showed the absence of growth for the bacteria after heat treatment and lactic acid addition. On the contrary, the addition of NaCl at all the concentrations tested and the UV irradiation did not affect the growth curves compared to the control. The oxygen deprivation limited the growth of bacteria.

## 4. Discussion

Pathogenic bacteria often have multiple temperate bacteriophages within their genome, which can be directed to a lytic cycle in response to a stress. During this work, different stressors related to the cheese making process were analysed to understand the extent of bacteriophage release. To study this phenomenon, 3 out of 35 STEC strains that showed the presence of an inducible prophage, were subjected to stressors.

Sodium chloride (NaCl) is added during cheese production to provide texture, consistency, crusting (frequently in ripened cheese varieties); to enhance the flavor, but mainly to inhibit microbial growth (Boisard et al., 2014). Salt content varies between 0.7 and 6.0%, depending on the type of cheese and the method of salting (Bansal and Mishra, 2020). Our findings are in accordance with Harris and colleagues (Harris et al., 2012) that observed increasing frequency in *stx2*+ prophage induction with NaCl concentration up to 2.0%, while 3% NaCl decreased prophage induction significantly.

Commonly, physical methods are used to inactivate pathogenic microorganisms, as well as spoilage microorganisms and their enzymes, ensuring food safety and extending shelf life. As already demonstrated, UV irradiation increased *stx1*-encoding-phage induction (Erill et al., 2007). Oxygen deprivation and pasteurization had no significant effect on induction compared to untreated cultures. Pasteurization treatment causes inactivation of STEC cultures, giving no possibility to any prophage induction event. The presence of phage-derived *stx-*1 genes reported in the samples can be related to a release that took place before treatment, during exponential growth. Given the adaptable metabolism of *E. coli*, oxygen deprivation should not pose substantial stress, favorable to prophage induction.

STEC strains were reported to sustain growth to a minimum of pH 4 for almost 8 h, after which their inherent acid resistance no longer prevented complete loss of viability (Molina et al., 2003). In our study, the bacteria were unable to duplicate under the proposed acidic conditions. Indeed, the pH of the medium after the addition of lactic acid was 3.42, 3.04 and 2.67 for 0.5, 1.5 and 3% of lactic acid (v/v) respectively. The acid-resistant phenotype of STEC is conjectured to be the chief determinant for their low infectious dose (Bernedo-Navarro and Yano, 2016). The reduction in prophage induction by lactic acid exposure was in line with a previous report that showed how stx induction was inhibited at a pH lower than 5.5 (Imamovic and Muniesa, 2012). Furthermore, in another study in which the phage release was evaluated by adding 1.5 and 3% of lactic acid, temperate phages could not be detected (Bonanno et al., 2017). This may be attributed to the negative effect of weak acids on STEC cellular viability: without significant changes to extracellular pH, assimilation of weak acids disrupts homeostasis by cytoplasmic acidification and anion accumulation, since the conjugate base is unable to diffuse out (Foster, 1999). Albeit significantly decreased, free phage particles in suspension were still detected. However, toxin transmission *via* transduction of viral DNA appears to be unlikely to succeed under acidic conditions. Lowering pH has a halting effect on capsid maturation of virion progeny (Lata et al., 2000), which should disable DNA packaging.

Bacterial growth was monitored through OD measurements after the addition of approximately Log10 6.2 UFC/mL into each tube prior to stress submission. As a result, the values obtained in pasteurization and lactic acid addition did not show any increase over time. However, in the case of NaCl 1.5 and 2%, phage release was significantly different but not observed in bacterial growth, comparing the curves to the control (Supplementary Figure S3).

In conclusion, some stressors related to cheese production can improve or decrease phage release, influencing the safety of the process. As previously reported (Bonanno et al., 2017) free phage particles could infect other *E. coli* both during cheese making process and in the human intestine after ingesting contaminated cheese, increasing the chance of infection. Furthermore, it can be considered that the growth phase of the bacteria influences the spontaneous release of the prophage: the exponential phase is the most favourable. Furthermore, the presence of free *stx-*phage could be a potential cause of false positives in food samples analysed by PCR (Bonanno et al., 2017).

## Data availability statement

The original contributions presented in the study are included in the article/supplementary material, further inquiries can be directed to the corresponding author.

## Author contributions

NM: Conceptualization, Formal analysis, Methodology, Software, Writing – original draft, Writing – review & editing. RV: Formal Analysis, Investigation. CP: Conceptualization, Data curation, Project administration, Supervision, Writing – review & editing.
