# Supplementary material for: Influence of cheese making process on STEC bacteriophage release
Source: Front Microbiol. 2023 Sep 28;14:1270346. doi: 10.3389/fmicb.2023.1270346 (PMC10569213; doi:10.3389/fmicb.2023.1270346)
Supplement: Supplementary file 1 [file Data_Sheet_1.docx]

Supplementary Material

Nicola Mangieri^1^, Rui P. Vieira^1,2^, Claudia Picozzi^1*^

^1^DeFENS, Department of Food, Environmental and Nutritional Sciences, Università degli Studi di Milano, Milano, Italy

^2^Instituto de Medicina Molecular, Faculdade de Medicina, Universidade de Lisboa, Lisboa, Portugal

*** Correspondence:**Claudia Picozzi
[claudia.picozzi@unimi.it](mailto:claudia.picozzi@unimi.it)

**Supplementary Figure 1.** Standard curve of 225R-A DNA from SYBR Green qPCR Log DNA copies/ml calculated from serial dilution from 1:10 to 1:100000.


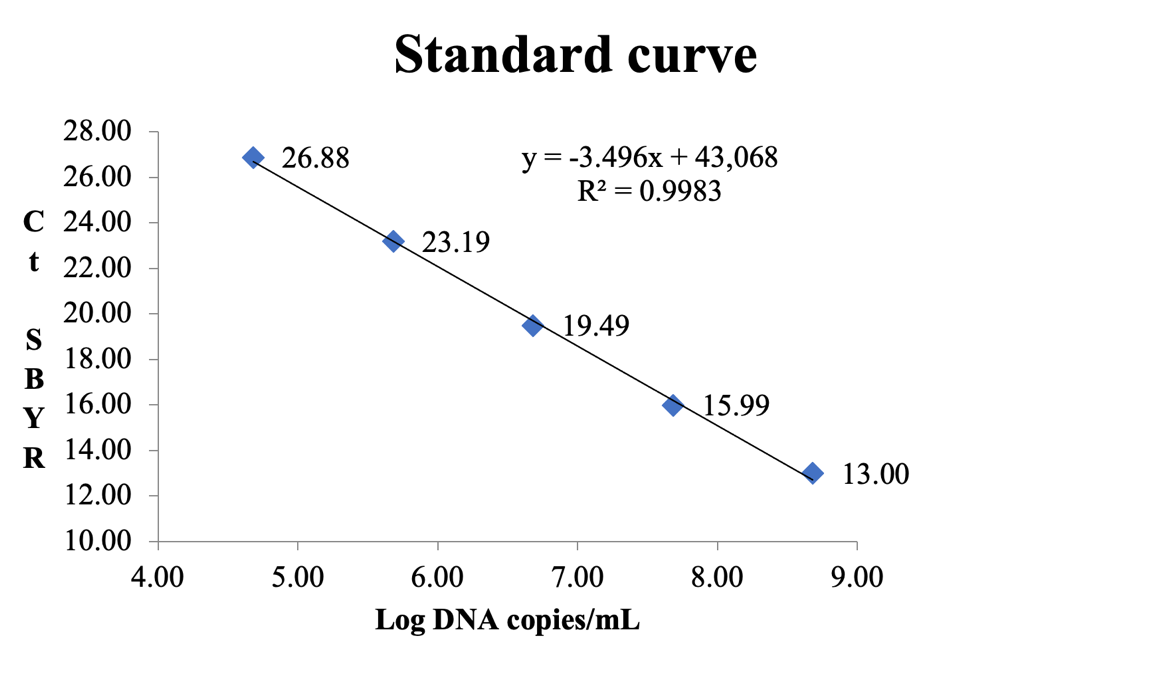


**Supplementary Figure 2.** Example of spot-test on LB agar plates for testing the presence of bacteriophage in the crude filtrates. 1: L36-2 crude lysate on (a) CNCTC 6246 and (b) CNCTC 6896; 2: F1-1 crude lysate on (a) CNCTC 6246 and (b) CNCTC 6896; 3: no crude lysate addition on (a) CNCTC 6246 and (b) CNCTC 6896.

**
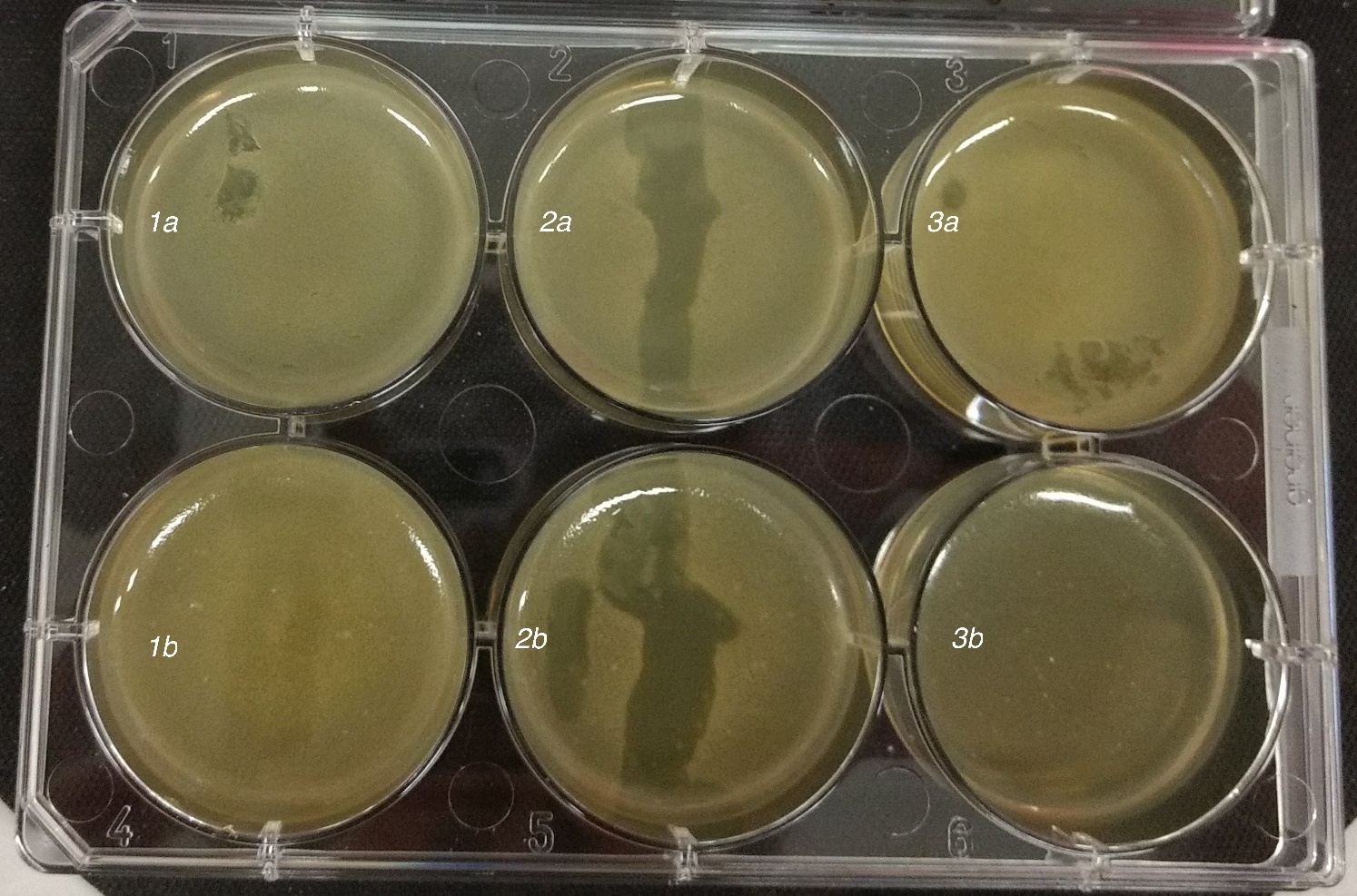
**

**Supplementary figure 3.** Bacterial growth of STEC strains subjected to different stressors.

**Supplementary Table 1.** Results of SYBR qPCR. Std: standard deviation; LacAc: Lactic acid; Ox: oxygen deprivation; T°: heat treatment; Sp: spontaneous release.

|  |  | **Log DNA copies/ml, replicates:** | | | |  |  |
| --- | --- | --- | --- | --- | --- | --- | --- |
| **bacteria** | **stressors** | **1** | **2** | **3** | **4** | **average** | **std** |
| 225R-A | 1%NaCl | 8.090 | 8.035 | 8.548 | 8.586 | 8.315 | 0.292 |
| 225R-A | 1.5%NaCl | 8.118 | 8.164 | 9.331 | 9.170 | 8.696 | 0.644 |
| 225R-A | 2%NaCl | 8.402 | 8.525 | 9.416 | 9.394 | 8.934 | 0.546 |
| 229RACH | 1%NaCl | 6.165 | 5.993 | 6.854 | 7.202 | 6.553 | 0.570 |
| 229RACH | 1.5%NaCl | 6.920 | 7.369 | 7.822 | 8.120 | 7.558 | 0.526 |
| 229RACH | 2%NaCl | 6.828 | 7.089 | 8.592 | 8.287 | 7.699 | 0.871 |
| F1-1 | 1%NaCl | 6.334 | 6.365 | 7.080 | 6.492 | 6.568 | 0.348 |
| F1-1 | 1.5%NaCl | 8.096 | 6.949 | 7.929 | 7.661 | 7.659 | 0.506 |
| F1-1 | 2%NaCl | 7.157 | 7.129 | 8.796 | 8.702 | 7.946 | 0.928 |
| 225R-A | UV | 8.402 | 8.673 | 8.570 | 8.787 | 8.608 | 0.164 |
| 229RACH | UV | 6.803 | 6.720 | 6.605 | 6.706 | 6.708 | 0.081 |
| F1-1 | UV | 7.017 | 6.868 | 7.030 | 7.011 | 6.981 | 0.076 |
| 225R-A | 0.5%LacAc | 5.747 | 5.776 | 5.543 | 5.495 | 5.640 | 0.142 |
| 225R-A | 1.5%LacAc | 5.618 | 5.481 | 4.904 | 4.860 | 5.216 | 0.390 |
| 225R-A | 3%LacAc | 5.641 | 5.475 | 5.093 | 5.222 | 5.358 | 0.247 |
| 229RACH | 0.5%LacAc | 5.507 | 5.701 | 5.030 | 4.980 | 5.305 | 0.355 |
| 22RACH | 1.5%LacAc | 5.467 | 5.424 | 5.024 | 4.845 | 5.190 | 0.305 |
| 229RACH | 3%LacAc | 5.561 | 5.561 | 4.892 | 5.483 | 5.374 | 0.324 |
| F1-1 | 0.5%LacAc | 5.524 | 5.656 | 4.911 | 4.873 | 5.241 | 0.407 |
| F1-1 | 1.5%LacAc | 5.421 | 5.530 | 4.820 | 4.804 | 5.144 | 0.386 |
| F1-1 | 3%LacAc | 5.533 | 5.387 | 4.908 | 4.999 | 5.206 | 0.301 |
| 225R-A | Ox | 7.495 | 7.535 | 7.599 | 7.523 | 7.538 | 0.044 |
| 229RACH | Ox | 5.942 | 5.867 | 5.473 | 6.662 | 5.986 | 0.495 |
| F1-1 | Ox | 6.700 | 6.565 | 6.391 | 6.476 | 6.533 | 0.132 |
| 225R-A | T° | 7.601 | 7.441 | 7.287 | 7.347 | 7.419 | 0.137 |
| 229RACH | T° | 5.481 | 5.558 | 5.002 | 4.986 | 5.257 | 0.305 |
| F1-1 | T° | 6.505 | 6.311 | 6.024 | 5.986 | 6.206 | 0.246 |
| 225R-A | Sp | 7.684 | 7.707 | 7.564 | 8.146 | 7.775 | 0.255 |
| 22RACH | Sp | 5.776 | 5.770 | 5.313 | 5.414 | 5.568 | 0.240 |
| F1-1 | Sp | 6.379 | 6.296 | 5.923 | 5.945 | 6.136 | 0.236 |
